# PCDH1 facilitates migration, proliferation, and stemness of pancreatic cancer cells through PI3K-Akt signaling

**DOI:** 10.3389/fonc.2025.1696695

**Published:** 2026-01-12

**Authors:** Zhiqiang Yang, Zhipeng Zhang, Ye Song, Chuang Dai, Weihui Zhang, Dali Zhao

**Affiliations:** 1 Department of General Surgery, The First Affiliated Hospital of Harbin Medical University, Harbin, Heilongjiang, China; 2 Key Laboratory of Hepatosplenic Surgery, Ministry of Education, The First Affiliated Hospital of Harbin Medical University, Harbin, China; 3 Department of Anorectal Surgery, The Fifth Hospital of Zunyi Medical University, Zhuhai, Guangdong, China

**Keywords:** gene, pancreatic ductal adenocarcinoma, PCDH1, stemness index, verification

## Abstract

**Background:**

Pancreatic ductal adenocarcinoma (PDAC) exhibits high lethality due to late diagnosis, therapy resistance, and cancer stem cell (CSC)-driven progression. However, the molecular determinants of stemness in PDAC remain unclear.

**Methods:**

We integrated RNA-sequencing and clinical data from TCGA to calculate stemness indices and identify hub genes using weighted co-expression network analysis and differential expression profiling. PCDH1 expression was validated in PDAC tissues by qRT-PCR and immunohistochemistry. Functional assays, including MTT, colony formation, Transwell, wound healing, flow cytometry, and spheroid culture, were performed following PCDH1 knockdown. Transcriptome sequencing was used to delineate downstream signaling mechanisms.

**Results:**

PCDH1 emerged as a stemness-associated oncogene strongly linked to poor survival. It was markedly overexpressed in PDAC tissues and cell lines. Silencing PCDH1 significantly impaired proliferation, clonogenicity, migration, and CSC frequency. Sphere-forming ability was also reduced. Transcriptomic analysis revealed downregulation of PI3K/AKT signaling upon PCDH1 knockdown, and rescue experiments confirmed its role in driving PDAC progression through PI3K/AKT activation. High PCDH1 expression further correlated with immune exclusion and distinctive patterns of chemosensitivity.

**Conclusion:**

PCDH1 is a novel stemness-linked oncogene that accelerates PDAC progression via PI3K/AKT signaling. Its prognostic relevance, association with immune evasion, and impact on drug responsiveness highlight PCDH1 as a promising biomarker and therapeutic target for CSC-directed intervention in PDAC.

## 1 Introduction

Pancreatic ductal adenocarcinoma (PDAC) represents one of the most devastating solid tumors, characterized by rapid progression, profound therapeutic resistance, and extremely poor survival outcomes (1). Despite advances in multimodal management, including surgery, cytotoxic chemotherapy, and immunotherapeutic approaches, clinical benefits remain modest and long-term survival is rare (2). The aggressive biology of PDAC arises not only from its mutational landscape but also from profound cellular plasticity and adaptation to environmental stressors, which collectively fuel recurrence and metastasis (3).

Mounting evidence indicates that a small subset of tumor cells endowed with stem-like features—commonly referred to as cancer stem cells (CSCs)—play a decisive role in sustaining tumor heterogeneity and therapeutic refractoriness (4). These cells possess enhanced self-renewal capacity, plasticity, and the ability to reconstitute tumor hierarchies, enabling them to survive conventional therapies and drive relapse (5). Consequently, the molecular underpinnings of CSC maintenance have become an area of intense investigation, as their disruption could attenuate malignant progression and improve therapeutic outcomes in PDAC (6, 7).

Among the pathways implicated in sustaining stemness, the PI3K/AKT signaling axis has emerged as a central regulator of tumor growth, metabolic adaptation, and resistance mechanisms (8, 9). Dysregulation of this pathway is frequently observed in pancreatic cancer and is closely associated with both disease aggressiveness and therapeutic failure (10, 11). Identifying upstream regulators that interface with PI3K/AKT signaling and confer stemness advantages could therefore reveal novel vulnerabilities amenable to therapeutic intervention.

Protocadherin-1 (PCDH1), a member of the protocadherin family, has recently been implicated in multiple pathological processes, but its role in pancreatic cancer biology remains largely unexplored (12). Given the growing recognition of cell adhesion molecules as regulators of stemness and microenvironmental interactions, investigating whether PCDH1 functions as a stemness-linked oncogene in PDAC holds substantial promise (13, 14). Here, we systematically interrogated transcriptomic datasets, validated expression patterns in patient specimens, and functionally characterized PCDH1 in PDAC models to determine its contribution to tumor progression and stemness regulation.

## 2 Methods and materials

### 2.1 Data acquisition

RNA-sequencing data and corresponding clinical annotations for 179 pancreatic ductal adenocarcinoma (PDAC) specimens and 4 adjacent normal pancreatic tissues were obtained from the TCGA-PAAD cohort. Raw expression data were downloaded in fragments per kilobase per million (FPKM) format, accompanied by patient-level metadata including overall survival, tumor grade, and stage. Data retrieval and preprocessing were performed through the UCSC Xena browser (http://www.genome.ucsc.edu/index.html), ensuring standardized normalization. Only samples with complete clinical and survival information were retained for downstream analysis, thereby minimizing bias from missing data.

### 2.2 Tumor stemness index

To quantify cellular dedifferentiation, we utilized stemness indices derived from multi-omics features, as established by Malta et al. (14). Both RNA-based (mRNAsi) and DNA methylation-based (mDNAsi) scores were applied to TCGA tumors, while additional stemness indices—RNAss and DNAss—were acquired from published resources. These indices reflect transcriptomic and epigenetic stemness, respectively. For PDAC samples, RNAss values were emphasized due to their stronger correlation with patient outcomes. The indices were subsequently integrated with clinical variables to assess their prognostic significance and biological relevance.

### 2.3 Survival analysis

Overall survival (OS) was defined as the primary clinical endpoint. Patients were stratified into high- and low-expression groups according to the median expression values of candidate genes or stemness indices. Kaplan–Meier survival curves were constructed, and differences were evaluated using log-rank tests. In addition, univariate Cox proportional-hazards regression was conducted to identify prognostic genes. Hazard ratios (HRs) with 95% confidence intervals (CIs) were calculated to estimate the relative risk of mortality. This dual approach ensured both statistical robustness and clinical interpretability.

### 2.4 Weighted gene co-expression analysis

To explore gene modules associated with stemness traits, we implemented WGCNA in R. Hierarchical clustering of all PDAC samples was performed to exclude outliers, followed by the determination of an appropriate soft-thresholding power (β) using the pickSoftThreshold function to approximate a scale-free network. Genes were subsequently organized into co-expression modules by dynamic tree cutting, and eigengenes for each module were correlated with stemness indices. Modules most strongly associated with RNAss were extracted for downstream analysis, thereby enriching for gene clusters potentially regulating stemness in PDAC.

### 2.5 Differential analysis

Differentially expressed genes (DEGs) between tumor and normal tissues were identified using the Limma package in R. Stringent thresholds of |log2 fold change| ≥ 1 and a false discovery rate (FDR) ≤ 0.05 were applied to ensure reliability. Volcano plots and heatmaps were generated to visualize transcriptional alterations, while significant DEGs were intersected with RNAss-associated WGCNA modules to obtain candidate hub genes for functional validation.

### 2.6 Protein-Protein Interaction network and enrichment analysis

Functional interactions among candidate genes were explored using the GeneMANIA platform (15), which integrates genetic, physical, and predicted interactions across multiple organisms. Constructed networks were used to visualize relationships between PCDH1 and its interacting partners, enabling insights into potential biological processes and pathways influenced by PCDH1 (15). To explore biological mechanisms underlying PCDH1 activity, ssGSEA was used to quantify enrichment scores for hallmark oncogenic pathways in TCGA-PDAC samples. Pearson correlations between PCDH1 expression and pathway scores were calculated, identifying signaling cascades significantly associated with PCDH1-driven tumor biology.

### 2.7 Immune infiltration profiling

To characterize the tumor immune microenvironment, single-sample gene set enrichment analysis (ssGSEA) was employed to estimate the relative abundance of 24 immune cell populations. Parallelly, the ESTIMATE algorithm was applied to derive immune, stromal, and ESTIMATE composite scores, reflecting global immune activity. For higher resolution, single-cell RNA-seq data from the TISCH database (16) were interrogated to evaluate PCDH1 expression across distinct tumor and immune subpopulations. This multi-level immune analysis enabled assessment of PCDH1 in immune regulation and tumor–host interactions (16).

### 2.8 Pharmacological sensitivity analysis

We employed the GSCALite platform to evaluate correlations between PCDH1 expression and therapeutic responsiveness. The Gene Set Differential Correlation (GSDC) module was used to assess pathway–drug interactions under varying conditions, while the Cancer Therapeutics Response Portal (CTRP) module predicted half-maximal inhibitory concentration (IC50) values for targeted agents and chemotherapeutics across diverse cell lines (17). These analyses allowed us to link PCDH1 expression with putative vulnerabilities to specific drugs.

### 2.9 Cell transfection and cell treatment

Normal pancreatic ductal epithelial cells (HPNE) and pancreatic cancer cell lines (HPAC, SW1990, PATU 8898t, CFPAC1, and BxPC3) were cultured in Dulbecco’s Modified Eagle Medium (DMEM) or RPMI-1640 supplemented with 10% fetal bovine serum (FBS) and 1% penicillin–streptomycin. Cultures were maintained in a humidified incubator at 37 °C with 5% CO_2_. Cell line authentication and mycoplasma testing were routinely performed to ensure experimental fidelity. Cells were introduced with either a negative control siRNA (si-NC) or a small interfering RNA specifically targeting PCDH1 (si-PCDH1), both synthesized by GENERAL BIOL (Anhui, China). Transfections were conducted using Lipofectamine 2000 (Life Technologies, CA, USA) following the manufacturer’s recommendations, and gene silencing efficiency was verified by qRT-PCR. After 48 h of incubation, sufficient suppression of PCDH1 was achieved, allowing cells to be used in subsequent functional assays. For rescue conditions, cells were treated with 100 ng/mL IGF-1 (HY-P7018, MedChemExpress) for 1 h. Immediately thereafter, proliferation, migration, and stemness-related assays were performed. All experiments were independently repeated in triplicate to ensure consistency and reliability of the results.

### 2.10 MTT assay

Twenty-four hours after transfection with 50 nM si-NC or si-PCDH1, BXPC3 and SW1990 cells were detached with 0.05% trypsin, counted, and seeded at 1×10³ cells per well in 96-well plates (100 µL complete medium + 10% FBS, five replicates). Cultures were incubated at 37 °C, 5% CO_2_ for 0, 24, 48, or 72 h; 20 µL MTT (5 mg/mL) was then added for 4 h. After removing the supernatant, 100 µL DMSO was added, plates were shaken for 10 min, and absorbance was read at 490 nm. Data are mean ± SD of three independent experiments (18).

### 2.11 Colony formation assay

Colony-forming capacity was assessed in BXPC3 and SW1990 cells 24 h after transfection with si-NC or si-PCDH1. Single-cell suspensions (300–500 cells/well, 6-well plate; density optimized per line) were cultured for 14–21 days under standard conditions with fresh complete medium supplied every 3–4 days. When colonies exceeded 50 cells they were gently washed with PBS, fixed in 4% paraformaldehyde (15 min, RT), stained with 0.1% crystal violet (15 min), rinsed and air-dried. Plates were scanned at 600 dpi and analyzed with ImageJ to determine colony number and mean diameter. All assays were performed in triplicate; results are presented as mean ± SD (19).

### 2.12 Transwell assay

Cell migration was assessed using 24-well Transwell chambers (Corning, NY, USA) equipped with polycarbonate membranes containing 8 μm pores. BXPC3 and SW1990 cells were transfected with si-NC or si-PCDH1 for 24 h, harvested with 0.05% trypsin, and resuspended in serum-free DMEM. Cell suspensions were adjusted to a final density of 1×10^4^ cells per 200 μL, which was seeded into the upper chambers. The lower chambers were filled with 600 μL of DMEM supplemented with 10% FBS to establish a chemoattractant gradient. Each condition was performed in triplicate to ensure reproducibility. Cells were incubated for 24 h under standard conditions (37 °C, 5% CO_2_, humidified atmosphere). After incubation, the non-migrated cells remaining on the upper surface of the membrane were gently removed using cotton swabs. Migrated cells adherent to the lower surface were washed twice with PBS, fixed in 4% paraformaldehyde for 15 min at room temperature, and stained with 0.1% crystal violet for 20 min. Excess dye was removed with PBS, and membranes were air-dried. Cells were visualized under an inverted microscope (Olympus, Japan) at 200× magnification, and migrated cells were quantified across five randomly selected fields per membrane. Migration results were averaged from three independent experiments and expressed as mean ± SD (20).

### 2.13 Wound healing assay

Cell migratory ability was further examined by a wound healing assay. BXPC3 and SW1990 cells were seeded in 6-well plates and grown to 90–95% confluence. To minimize proliferation-related bias, cells were starved in serum-free medium for 12 h prior to scratching. A uniform scratch was generated across the monolayer using a sterile 200 μL pipette tip held perpendicular to the plate. Detached cells were gently washed away with PBS, and fresh serum-free DMEM was added to maintain quiescence. Images of the wound were captured at 0, 24, and 48 h using phase-contrast microscopy (Leica, Germany), ensuring identical locations were imaged by marking reference points on the plate underside (21).

### 2.14 Immunohistochemistry

Formalin-fixed, paraffin-embedded (FFPE) pancreatic ductal adenocarcinoma (PDAC) tissues and matched adjacent normal pancreatic tissues were obtained from six patients who underwent surgical resection at the First Affiliated Hospital of Harbin Medical University, following informed consent and institutional ethical approval. Tissue sections (3 μm) were cut using a rotary microtome, mounted on glass slides, baked at 60 °C for 1 h, and sequentially deparaffinized in xylene and rehydrated through graded ethanol solutions. Antigen retrieval was carried out in citrate buffer (pH 6.0) using microwave heating for 15 min. Endogenous peroxidase activity was quenched with 3% hydrogen peroxide for 10 min at room temperature, and non-specific binding was blocked with 5% bovine serum albumin for 30 min. Slides were incubated overnight at 4 °C with a rabbit polyclonal anti-PCDH1 primary antibody (bs-111111R, Bioss Biotechnology, China) at the manufacturer-recommended dilution. After PBS rinsing, sections were incubated with a horseradish peroxidase (HRP)-conjugated secondary IgG antibody (PV-9000, ZSGB-BIO, China) for 20 min at room temperature. Visualization was performed using a diaminobenzidine (DAB) substrate, and nuclei were counterstained with hematoxylin. Negative controls were prepared by substituting primary antibodies with isotype controls. Stained sections were imaged under a bright-field microscope at 400× magnification. For each specimen, three randomly selected high-power fields were captured. Quantitative assessment of PCDH1 expression was performed using Image-Pro Plus (IPP) software, measuring integrated optical density (IOD) and normalizing against the stained area to yield mean optical density (MOD). All analyses were conducted in duplicate by two independent investigators blinded to the clinical diagnosis to minimize observer bias.

### 2.15 Sphere formation assay

The self-renewal ability of pancreatic cancer cells was assessed by a sphere formation assay under non-adherent, serum-free conditions. BXPC3 and SW1990 cells were enzymatically dissociated into single-cell suspensions, and viable cells were counted using trypan blue exclusion. The concentration was adjusted to 1.5×10^4^ cells/mL, and 1 mL of this suspension was seeded into 24-well ultra-low attachment plates (Corning, USA) for pre-culture. Subsequently, cells were diluted to 4×10³ cells/well in 200 μL and plated into U-bottom 96-well ultra-low attachment plates, ensuring nine replicate wells per condition. To reduce edge effects from evaporation, 200 μL of sterile PBS was added to peripheral wells. Cells were cultured at 37 °C in a humidified atmosphere with 5% CO_2_, in serum-free DMEM/F12 medium supplemented with 20 ng/mL epidermal growth factor (EGF), 20 ng/mL basic fibroblast growth factor (bFGF), and B27 supplement (1:50, Gibco). Medium was replenished every 3 days. Photographs were captured at 0, 24, and 48 h, and again on day 5, using an inverted phase-contrast microscope at 40× magnification. Sphere number and diameter were quantified using ImageJ software. A minimum cutoff of 50 μm diameter was applied to define valid spheres. For each experimental group, nine wells were analyzed, and results were averaged across three independent experiments. Data were expressed as mean ± SD, and statistical significance of group differences was evaluated by Student’s t-test (22).

### 2.16 Flow cytometry analysis

Flow cytometry was employed to evaluate the expression of stemness-associated surface markers in PDAC cells. After transfection and appropriate culture treatments, cells were harvested by gentle trypsinization, washed twice in cold phosphate-buffered saline (PBS), and resuspended in staining buffer (PBS containing 2% fetal bovine serum) at a concentration of 1×10^6^ cells/mL. Cells were aliquoted into 1.5 mL Eppendorf tubes according to experimental groups, ensuring equal cell counts across conditions. For surface antigen staining, cells were incubated with fluorochrome-conjugated monoclonal antibodies, including anti-CD133 (12-1338-42, Thermo Fisher) and anti-CD24 (11-0247-42, Thermo Fisher), at manufacturer-recommended dilutions. CD24 conjugates with FITC, CD133 conjugates with PE. Parallel tubes containing isotype-matched control antibodies were prepared to account for non-specific binding. Staining was performed for 30 min at 4 °C in the dark to prevent fluorochrome degradation, with gentle vortexing every 10 min to ensure uniform suspension and antibody access. After incubation, cells were washed twice with cold PBS to remove unbound antibodies and resuspended in 500 μL of staining buffer for analysis. To exclude dead cells, propidium iodide (PI) was added immediately before acquisition when required. Data acquisition was performed on a FACSCalibur™ flow cytometer (BD Biosciences, San Jose, USA) equipped with a 488 nm argon laser. A minimum of 1×10^4^ events were collected per sample, and compensation settings were adjusted using single-stained controls. Data analysis was conducted using FlowJo software (Tree Star Inc., Ashland, OR, USA). Marker-positive cell populations were gated based on forward/side scatter and isotype controls. Final results were reported as the percentage of CD133^+^ or CD24^+^ cells relative to the total viable population. All experiments were repeated independently at least three times.

### 2.17 Statistical analysis

All statistical analyses were performed using R software (version 4.2.0) and GraphPad Prism (version 9.0) to ensure robustness. Data were first tested for normality using the Shapiro–Wilk test. For normally distributed variables, differences between two groups were assessed using Student’s t-test; otherwise, the Mann–Whitney U test was applied. For comparisons among multiple groups, one-way ANOVA or the Kruskal–Wallis test was used as appropriate. Categorical variables were compared with chi-squared or Fisher’s exact tests. Correlations between continuous variables were evaluated using Spearman’s rank correlation analysis. Levels of significance were denoted as P < 0.05(*), P < 0.01(**), P < 0.001 (***), and P < 0.0001 (****), with “ns” indicating non-significance.

## 3 Results

### 3.1 Association of stemness indices with prognosis and identification of RNAss-related gene modules

**Figure 1:**
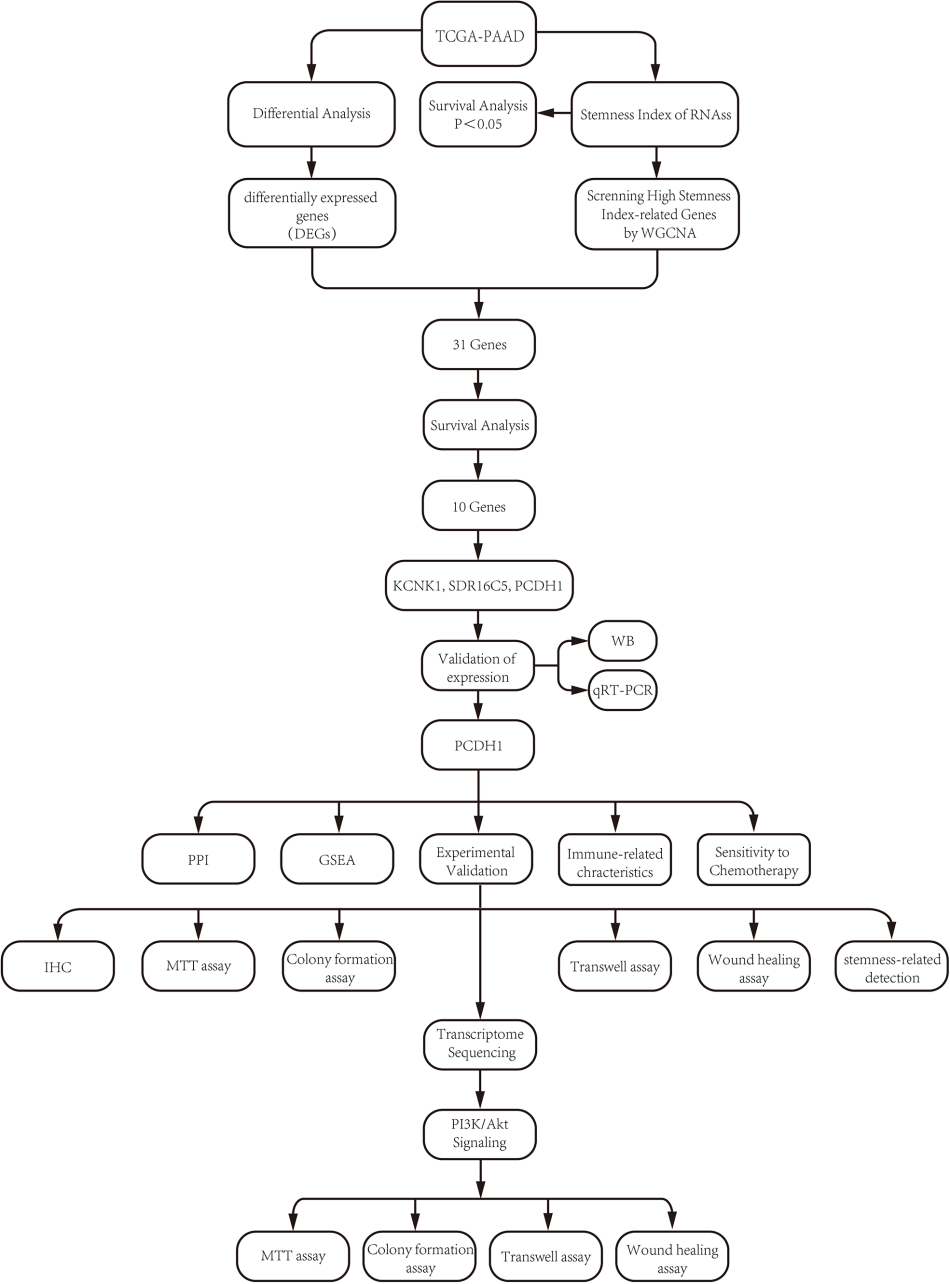
Flow chart.

**Figure 2:**
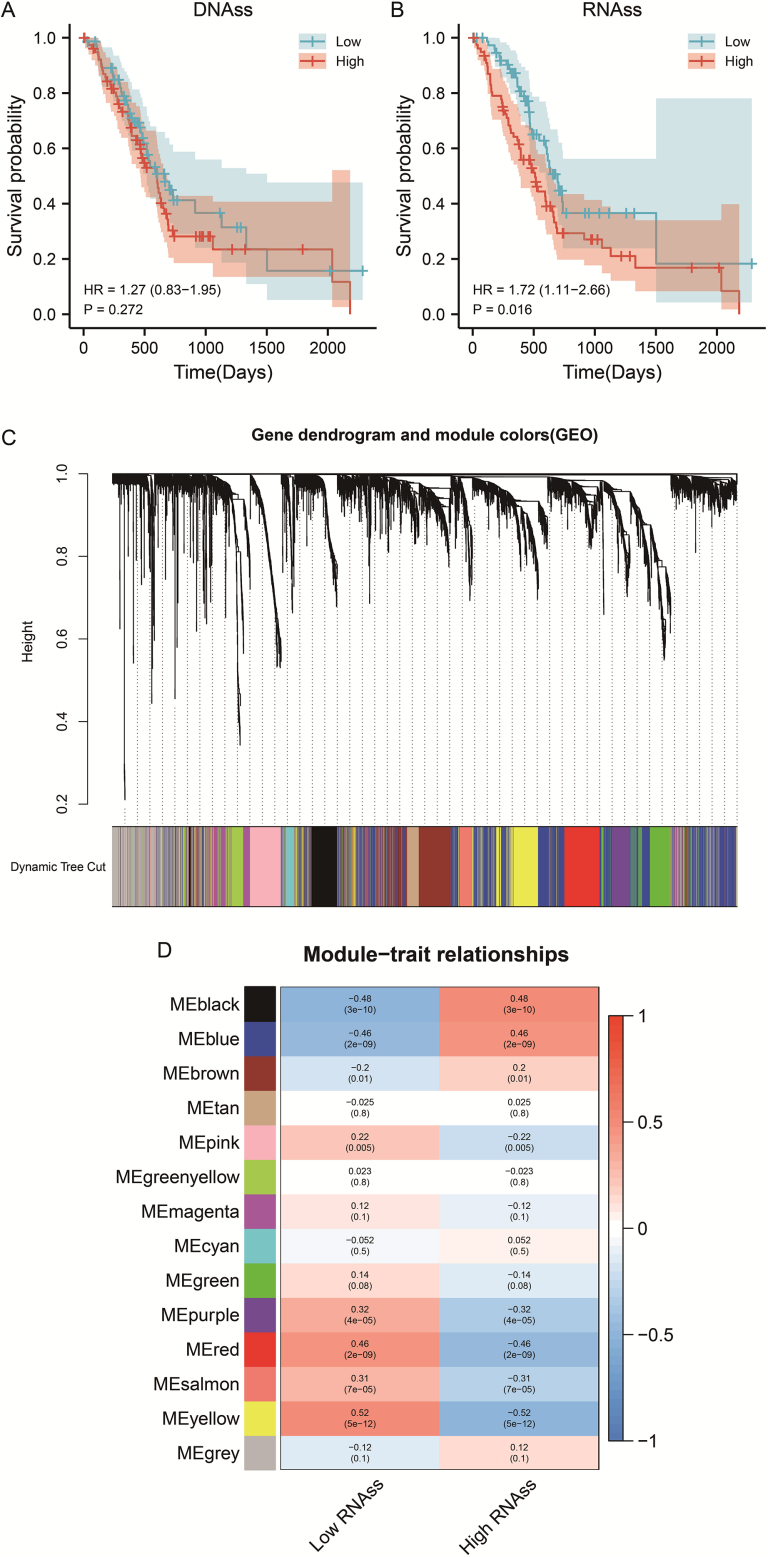
WGCNA screened the gene modules with significant positive correlation with RNAss index. **(A)** Survival curve of PDAC patients with different DNAss index. **(B)** Survival curve of PDAC patients with different RNAss index. **(C)** Gene dendrogram and module colors (GEO). **(D)** Module-trait relationships.

The overall research workflow is illustrated in **Figure 1**. To assess the prognostic relevance of stemness in PDAC, patients were stratified according to DNA methylation–based stemness index (DNAss) and RNA expression–based stemness index (RNAss). Kaplan–Meier survival analysis revealed no significant association between DNAss and overall survival (OS) (HR = 1.27, 95% CI 0.83–1.95, P = 0.272; **Figure 2A**). In contrast, patients with elevated RNAss exhibited markedly poorer OS compared with those in the low RNAss group (HR = 1.72, 95% CI 1.11–2.66, P = 0.016; **Figure 2B**), underscoring the clinical impact of transcriptional stemness in PDAC. To further explore molecular correlates of RNAss, we constructed a weighted gene co-expression network (WGCNA). Hierarchical clustering identified 14 distinct gene modules, each represented by a unique color (**Figure 2C**). Module–trait correlation analysis demonstrated that the black (r=0.48, P = 3e–10) and blue (r=0.46, P = 2e–09) modules showed the strongest positive associations with RNAss, suggesting that genes within these modules may contribute to stemness-associated tumor biology (**Figure 2D**).

### 3.2 Identification of RNAss-related hub genes in PDAC

**Figure 3:**
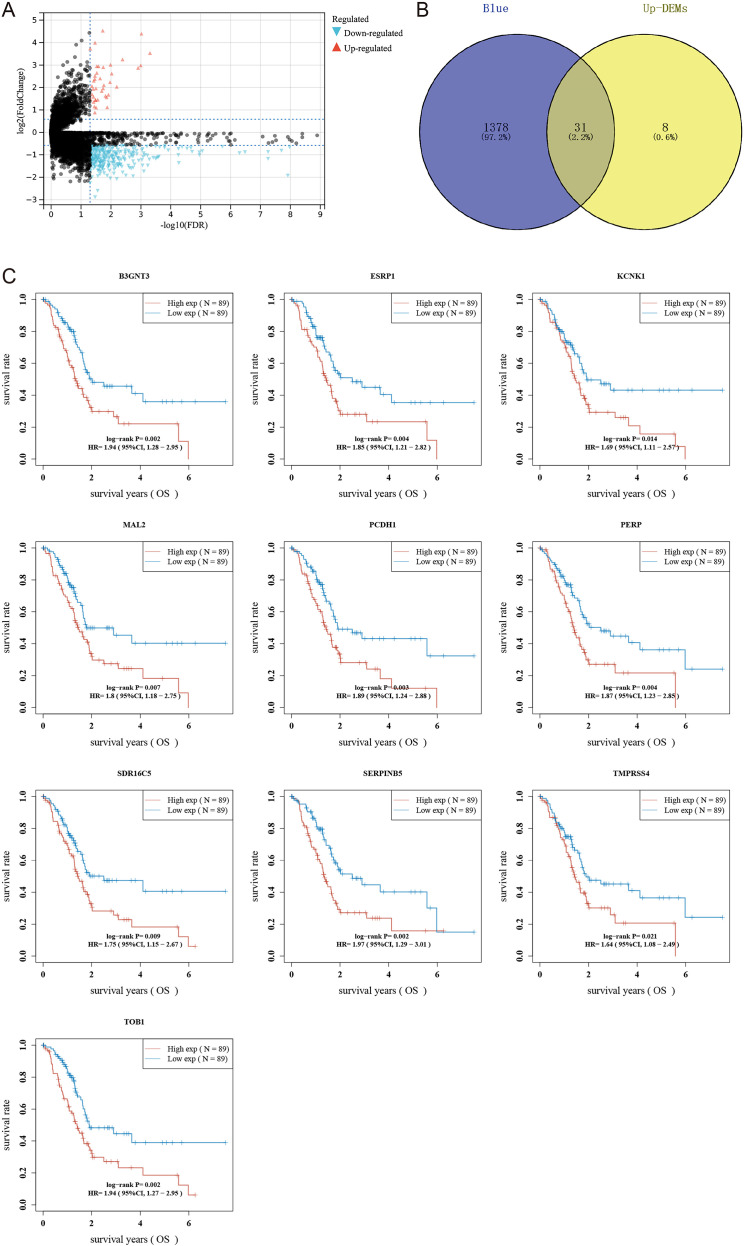
Screening hub genes related to RNAss index. **(A)** Volcano map shows differentially expressed genes in PDAC. **(B)** Wayne diagram intersects blue module, black module, and differentially expressed genes. **(C)** Survival curves of 10 hub genes.

To pinpoint hub genes associated with stemness and tumor development, we analyzed the overlap between the 39 upregulated differentially expressed genes (DEGs) and the 1,687 genes from the RNAss-related black and blue modules. This comparison revealed 31 common genes (**Figures 3B**), all showing elevated expression in PDAC tissues and a positive correlation with RNAss. Subsequently, we conducted univariate Cox regression to evaluate the prognostic value of these genes, identifying ten with significant links to overall survival (OS) in PDAC: B3GNT3, ESRP1, KCNK1, MAL2, PCDH1, PERP, SDR16C5, SERPINB5, TMPRSS4, and TOB1. For further experimental validation, we focused on the least explored genes in PDAC research based on literature review. Among these, KCNK1, SDR16C5, and PCDH1 emerged as understudied candidates, warranting deeper investigation.

### 3.3 Expression patterns and cellular localization of RNAss-associated hub genes

**Figure 4:**
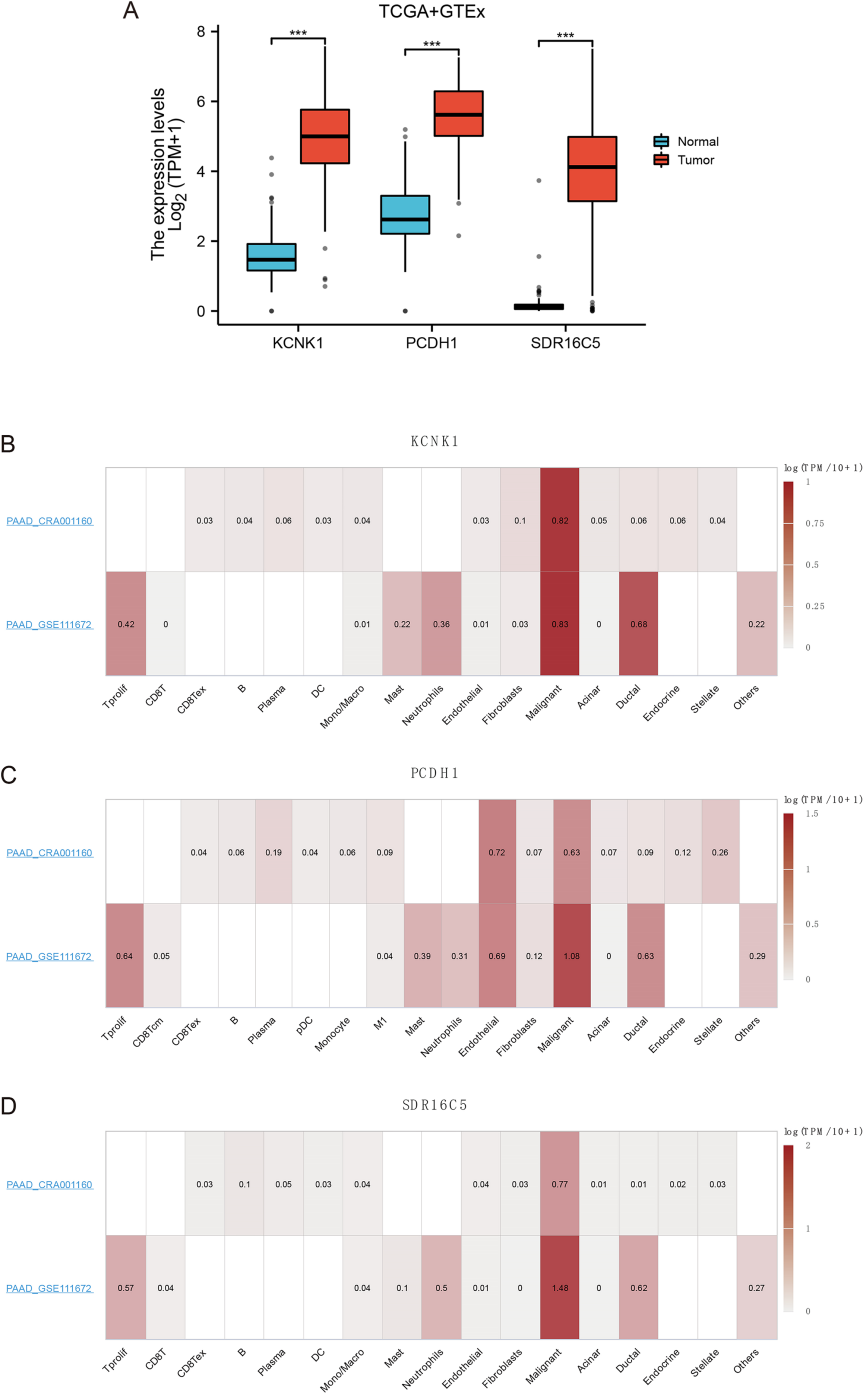
Expression of 3 hub genes **(A)** Gene expression of KCNK1, PCDH1 and SDR16C5. **(B)** Expression of KCNK1 in a single cell. **(C)** Expression of PCDH1 in a single cell. **(D)** Expression of SDR16C5 in a single cell.

To further validate candidate stemness-associated genes, we compared the expression of KCNK1, PCDH1, and SDR16C5 between PDAC and normal pancreatic tissues using TCGA and GTEx datasets. All three genes were significantly upregulated in tumors relative to non-malignant samples (**P<0.001; **Figure 4A**), suggesting potential oncogenic roles in PDAC. We next examined their cellular distribution using single-cell transcriptomic datasets (PAAD_CRA001160 and PAAD_GSE111672). KCNK1 expression was predominantly enriched in malignant epithelial cells, with additional signals in ductal cells and fibroblasts (**Figure 4B**). PCDH1 displayed broader localization, being highly expressed in malignant cells as well as fibroblasts, endothelial cells, and select immune subsets, consistent with a role in mediating tumor–stroma interactions (**Figure 4C**). SDR16C5 was strongly enriched in malignant populations and acinar cells, but largely absent from immune or stromal compartments (**Figure 4D**). Together, these findings confirm that KCNK1, PCDH1, and SDR16C5 are aberrantly upregulated in PDAC and exhibit distinct yet complementary patterns of cellular distribution, implicating them as potential contributors to tumor progression. Notably, the broad expression of PCDH1 across malignant and stromal cell types highlights it as a particularly compelling candidate for functional validation.

### 3.4 Validation and clinical relevance of PCDH1 expression

**Figure 5:**
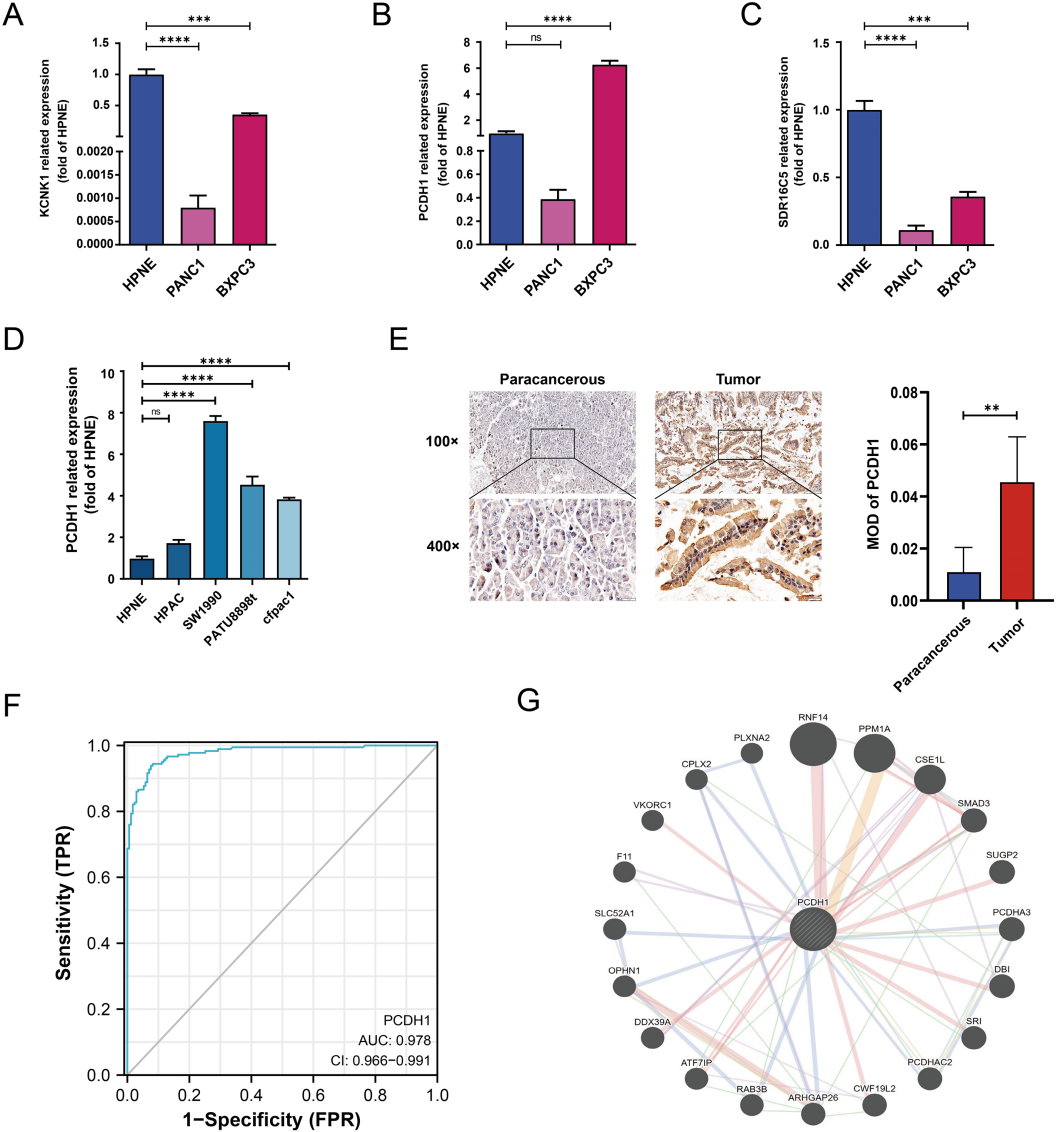
Related assays on the expression of hub genes. **(A–C)** mRNA expression of KCNK1, PCDH1 and SDR16C5 in HPNE and BXPC3. **(D)** mRNA expression of PCDH1 in HPNE and multiple PDAC cell lines. **(E)** Matched pairs of PDAC tissues and adjacent non-neoplastic pancreatic tissues were stained with PCDH1-specific antibodies for immunohistochemical analysis. Representative images of tissues and tumor-infiltrating cells are shown (100×, scale bar = 100 μm; 400×, scale bar = 20 μm). The MOD of PCDH1 is obtained by analyzing the photo-optical density with IPP software. **(F)** ROC curve of PCDH1 in PDAC. **(G)** Protein-protein interaction network of PCDH1. (ns: *P*>0.05, *P < 0.05, **P < 0.01, ***P < 0.001, ****P < 0.0001).

We next validated the expression of candidate stemness-associated genes in PDAC cell lines and patient tissues. qRT-PCR analysis confirmed that KCNK1 and SDR16C5 were downregulated in PANC-1 but moderately expressed in BxPC3 relative to normal pancreatic epithelial cells (HPNE) (**Figures 5A, C**). In contrast, PCDH1 displayed marked upregulation in BxPC3, while expression remained low in PANC-1 (**Figure 5B**). Expanded profiling across additional PDAC lines revealed consistently elevated PCDH1 expression in SW1990, PATU8988, and CFPAC1 cells compared to HPNE, whereas HPAC exhibited relatively modest levels (**Figure 5D**). Immunohistochemistry of paired tumor and adjacent tissues from six patients further demonstrated pronounced membranous PCDH1 staining in PDAC specimens compared with paracancerous counterparts. Quantitative analysis confirmed significantly higher mean optical density (MOD) in tumor tissues (P<0.01; **Figure 5E**). Receiver operating characteristic (ROC) analysis showed that PCDH1 expression had strong discriminatory power for distinguishing PDAC from normal tissue, with an area under the curve (AUC) of 0.978 (95% CI: 0.966–0.991; **Figure 5F**). To explore molecular interactions, a protein–protein interaction (PPI) network centered on PCDH1 was constructed. This analysis revealed associations with multiple proteins, including SMAD3, RNF14, PPM1A, and OPHN1, implicating PCDH1 in signaling cascades relevant to tumor progression and stemness regulation (**Figure 5G**). Collectively, these findings confirm that PCDH1 is robustly overexpressed in PDAC at both cellular and tissue levels, possesses high diagnostic value, and is embedded within oncogenic signaling networks, supporting its role as a candidate stemness-linked oncogene. Following closely, we further explored the correlation between clinical information and PCDH1 levels in PDAC patients in the TCGA cohort. The outcomes revealed that PCDH1 displayed a degree of variability in pathological grading, which means its expression was significantly higher in patients with G2 & G3 & G4 grading compared to those with G1 grading (**Table 1**).

**Table 1:** Relationship between clinical information and PCDH1 expression in PDAC patients of TCGA.

Characteristics	Total(N)	Odds Ratio(OR)	*P* value
T stage (T3&T4 vs. T1)	152	1.429 (0.305-7.465)	0.648
N stage (N1 vs. N0)	173	0.646 (0.330-1.250)	0.197
M stage (M1 vs. M0)	84	3.524 (0.494-70.590)	0.269
Pathologic stage (Stage II & Stage III & Stage IV vs. Stage I)	175	1.803 (0.718-4.787)	0.217
Gender (Male vs. Female)	178	1.578 (0.873-2.873)	0.133
Age (>65 vs. <=65)	178	1.046 (0.580-1.886)	0.881
Histologic grade (G2&G3&G4 vs. G1)	176	4.463 (1.892-11.818)	0.001
Smoker (Yes vs. No)	144	0.836 (0.432-1.612)	0.593
Alcohol history (Yes vs. No)	166	0.808 (0.431-1.507)	0.503
History of diabetes (Yes vs. No)	146	0.805 (0.382-1.689)	0.566
History of chronic pancreatitis (Yes vs. No)	141	2.321 (0.717-8.921)	0.179
Family history of cancer (Yes vs. No)	110	0.704 (0.328-1.501)	0.364

### 3.5 PCDH1 expression is associated with immune infiltration in PDAC

**Figure 6:**
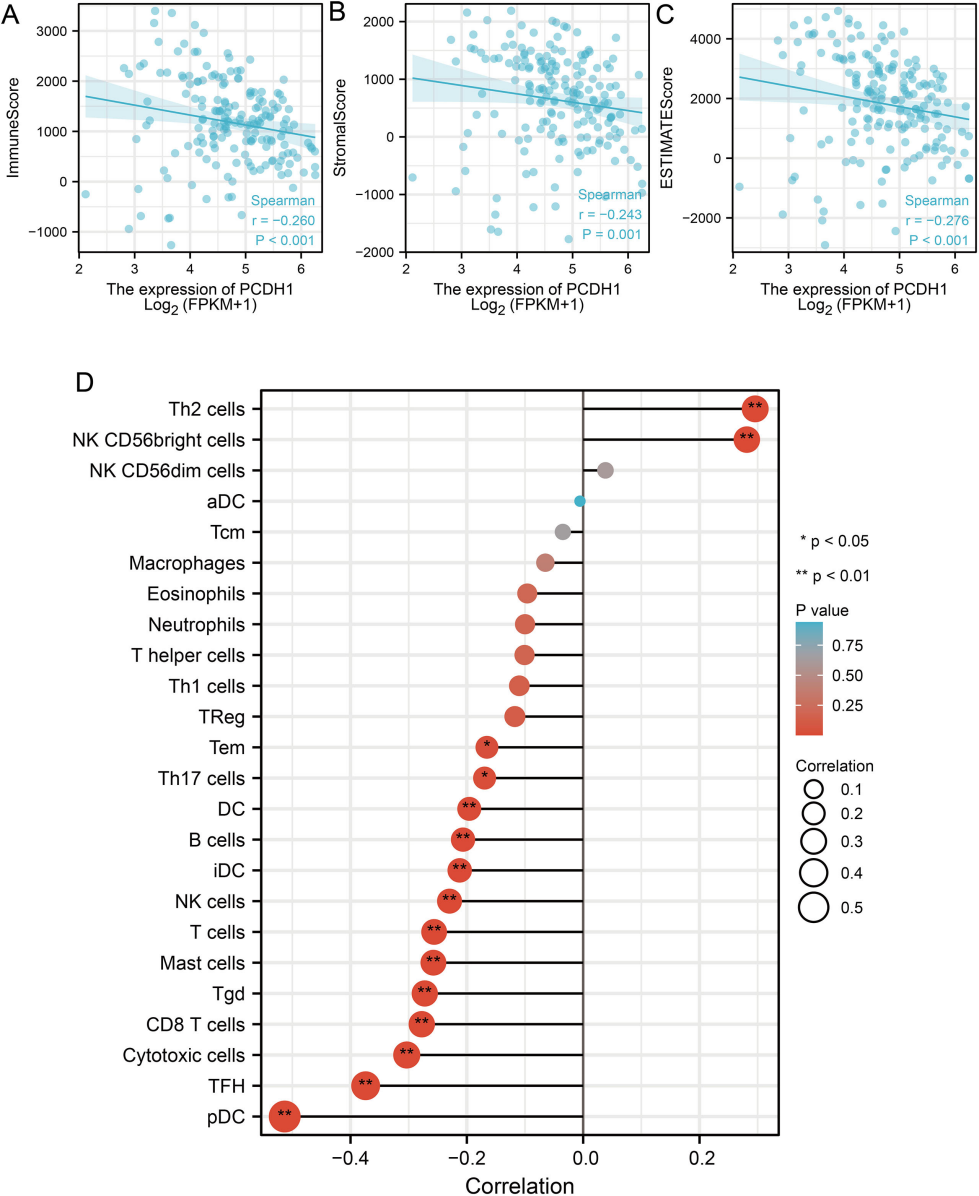
Correlation between PCDH1 and immune infiltration. **(A)** Correlation between PCDH1 and immune score. **(B)** Correlation between PCDH1 and stromal score. **(C)** Correlation between PCDH1 and ESTIMATE score. **(D)** Correlation between PCDH1 and immune cells. (*P < 0.05, **P < 0.01).

To further elucidate the immunological role of PCDH1 in PDAC, we evaluated its association with tumor immune infiltration using ESTIMATE and CIBERSORT algorithms. Elevated PCDH1 expression was significantly correlated with reduced immune infiltration, as indicated by negative correlations with ImmuneScore (r = –0.260, P<0.001; **Figure 6A**), StromaScore (r = –0.243, P = 0.001; **Figure 6B**), and ESTIMATEScore (r = –0.276, P<0.001; **Figure 6C**). Detailed immune cell profiling revealed that PCDH1 expression was inversely associated with multiple effector populations, including plasmacytoid dendritic cells (pDCs), follicular helper T (TFH) cells, cytotoxic T cells, CD8^+^ T cells, and regulatory T cells (Tregs), as well as B cells and antigen-presenting subsets (**Figure 6D**). Conversely, its correlations with Th2 cells and NK cell subsets were weak or nonsignificant. These findings suggest that PCDH1 overexpression may contribute to an immunosuppressive tumor microenvironment in PDAC, characterized by reduced infiltration of adaptive immune cells and impaired antitumor immunity.

### 3.6 Association of PCDH1 expression with drug sensitivity

**Figure 7:**
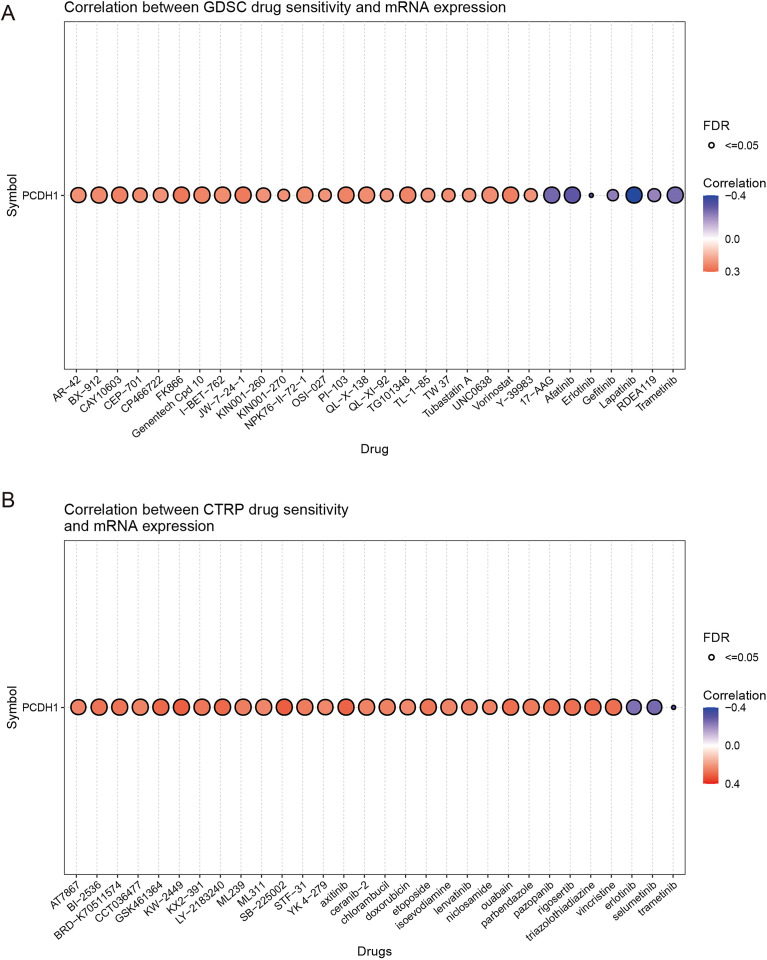
Chemosensitivity of PCDH1. **(A)** Correlation between GDSC drug sensitivity and PCDH1 mRNA expression **(B)** Correlation between CTRP drug sensitivity and PCDH1 mRNA expression.

To explore the potential therapeutic implications of PCDH1, we assessed its correlation with drug sensitivity using the GDSC and CTRP pharmacogenomic datasets. In the GDSC cohort, high PCDH1 expression was significantly associated with increased sensitivity to a broad spectrum of chemotherapeutic and targeted agents, including gemcitabine, paclitaxel, and several kinase inhibitors (correlation coefficients ≈0.3–0.4, FDR ≤ 0.05; **Figure 7A**). In contrast, negative correlations were observed with selected agents, most notably Erlotinib, Lapatinib, and Trametinib (r≈–0.4, FDR ≤ 0.05), suggesting potential resistance mechanisms linked to PCDH1 overexpression. Similarly, CTRP analysis corroborated these findings, demonstrating widespread positive correlations between PCDH1 expression and sensitivity to multiple cytotoxic drugs and small-molecule inhibitors. Conversely, resistance associations were again evident with a subset of tyrosine kinase inhibitors, including Sorafenib and Lapatinib (**Figure 7B**). Collectively, these pharmacogenomic data indicate that PCDH1 expression may serve as a predictive biomarker of drug response in PDAC, conferring differential sensitivity across distinct therapeutic classes. This dual role highlights the need for context-dependent evaluation of PCDH1 when considering treatment strategies.

### 3.7 Functional effects of PCDH1 knockdown in PDAC cells

**Figure 8:**
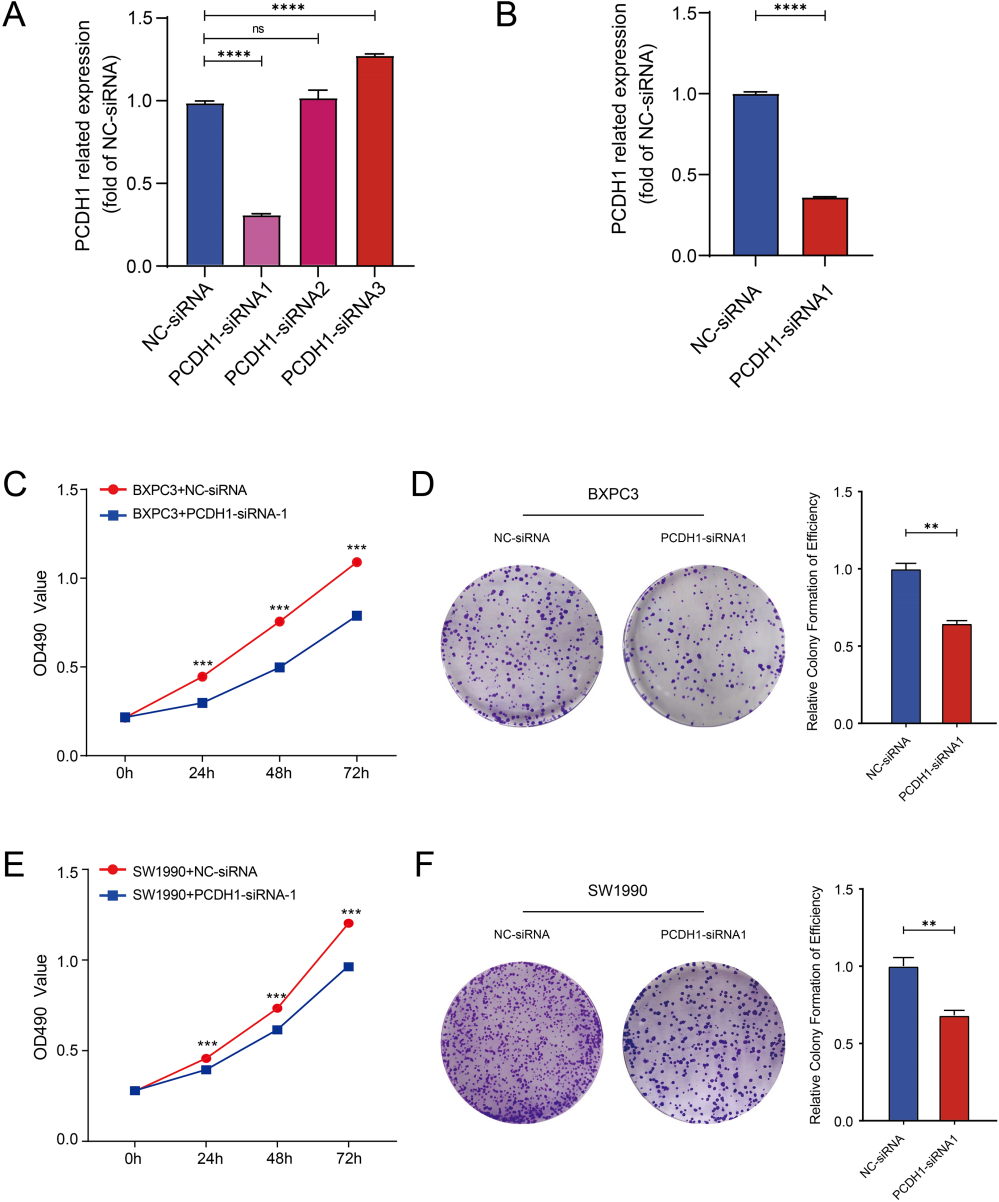
Knockdown of PCDH1 inhibited the proliferation of PDAC. **(A)** Efficiency of knocking down mRNA of PCDH1 si-RNA in BXPC3. **(B)** Efficiency of knocking down mRNA of PCDH1 si-RNA in SW1990. **(C, E)** MTT assays reveal the proliferation ability of control group and PCDH1 knockdown group in cell line BXPC3 and SW1990. **(D, F)** Colony formation assays reveal the proliferation ability of control group and PCDH1 knockdown group in cell line BXPC3 and SW1990. (ns: *P*>0.05, *P < 0.05, **P < 0.01, ***P < 0.001, ****P < 0.0001).

To experimentally validate the oncogenic role of PCDH1, we performed loss-of-function assays in PDAC cell lines. Among three designed siRNAs, PCDH1-siRNA1 exhibited the highest silencing efficiency as determined by qRT-PCR, and was therefore selected for subsequent experiments (**Figures 8A, B**). MTT assay demonstrated that PCDH1 knockdown significantly suppressed cell proliferation in both BxPC3 and SW1990 cells across multiple time points (P<0.001; **Figures 8C, E**). Consistently, colony formation assays revealed that the clonogenic capacity of PCDH1-deficient cells was markedly reduced compared with control groups (**Figures 8D, F**). These findings collectively indicate that PCDH1 promotes PDAC cell growth and clonogenic survival, further substantiating its role as a functional oncogene and a potential therapeutic target.

### 3.8 PCDH1 depletion suppresses migratory capacity of PDAC cells

**Figure 9:**
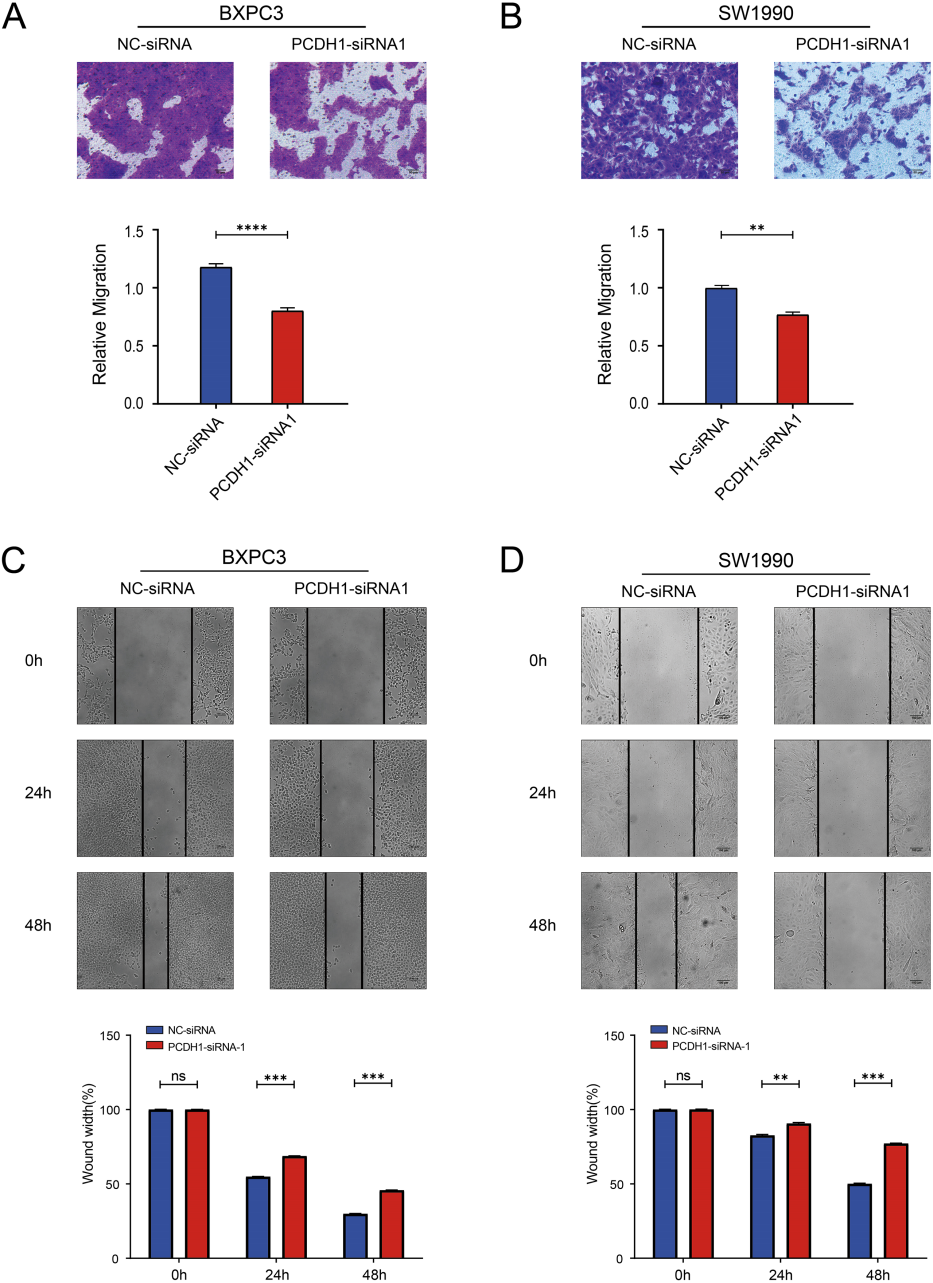
Knockdown of PCDH1 inhibited the migration of PDAC. **(A)** Transwell assays show the migration ability of the control group and the PCDH1 knockdown group in the BXPC3 cell line. (Scale bar = 50μm) **(B)** Transwell assays show the migration ability of the control group and the PCDH1 knockdown group in the SW1990 cell line. (Scale bar = 50μm) **(C)** Wound healing assays exhibit the migration ability of the control group and the PCDH1 knockdown group in the BXPC3 cell line. (Scale bar = 100μm) **(D)** Wound healing assays exhibit the migration ability of the control group and the PCDH1 knockdown group in the SW1990 cell line. (Scale bar = 100μm) (ns: *P*>0.05, *P < 0.05, **P < 0.01, ***P < 0.001, ****P < 0.0001).

To assess the impact of PCDH1 on tumor cell motility, we performed both Transwell migration and wound-healing assays in BxPC3 and SW1990 cells. Transwell analysis demonstrated that silencing PCDH1 markedly reduced the number of migrated cells compared with negative control groups (**Figures 9A, B**). Consistently, wound-healing assays revealed significantly delayed closure of scratch gaps in PCDH1-deficient cells. In both BxPC3 and SW1990 models, the migratory index was significantly reduced at 24 h and 48 h following knockdown, indicating impaired collective migration (**Figures 9C, D**). Together, these findings provide functional evidence that PCDH1 promotes the migratory phenotype of PDAC cells, supporting its role in driving tumor invasiveness.

### 3.9 PCDH1 knockdown attenuates stemness phenotypes in PDAC cells

**Figure 10:**
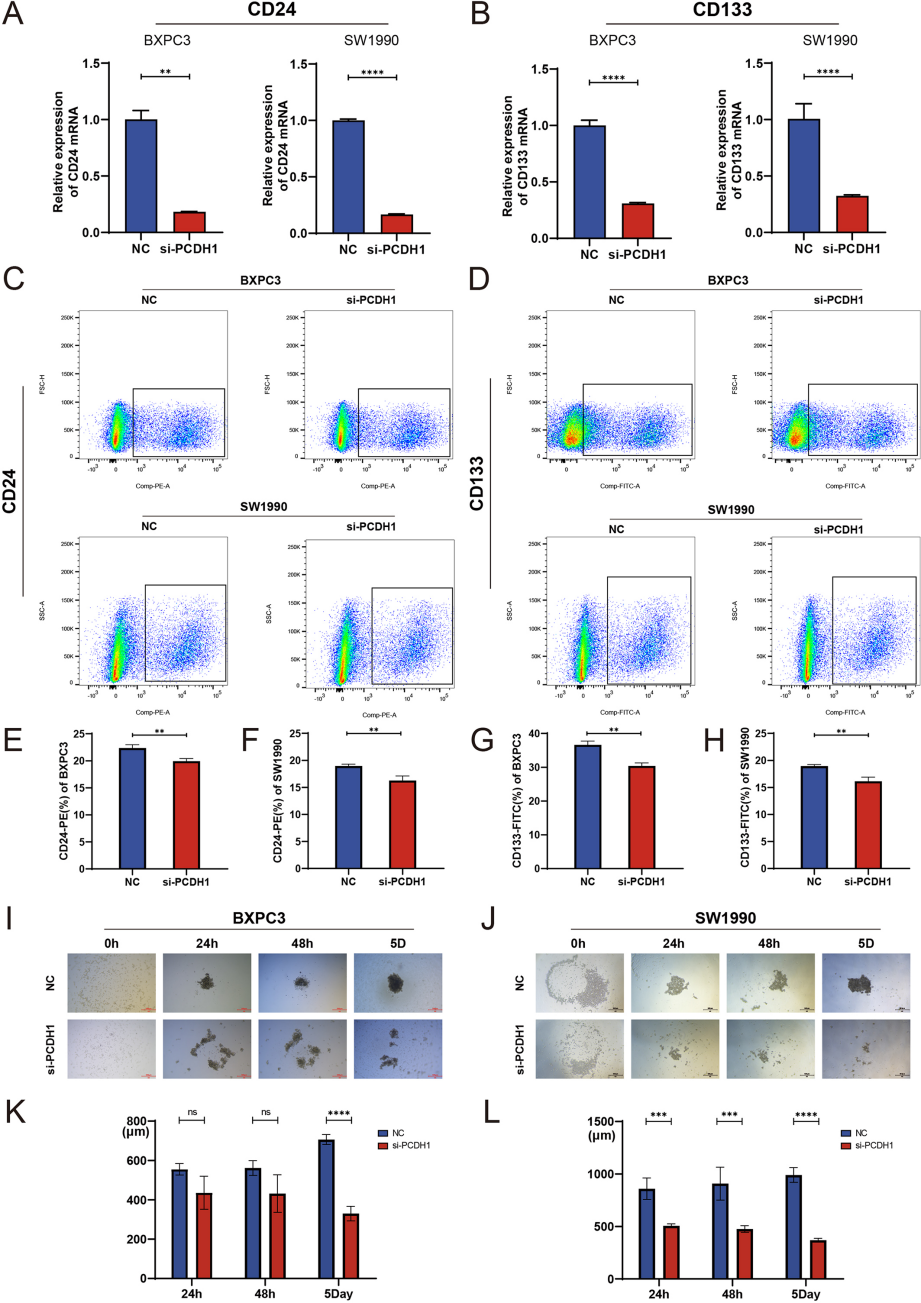
Knockdown of PCDH1 inhibited the tumor stemness of PDAC cell lines. **(A)** Relative expression of CD24 mRNA in the control group and the PCDH1 knockdown group. **(B)** Relative expression of CD133 mRNA in the control group and the PCDH1 knockdown group. **(C)** Detection of CD24^+^ cells in the control and PCDH1 knockdown groups via flow cytometry in BXPC3 and SW1990 cell lines. **(D)** Detection of CD133^+^ cells in the control and PCDH1 knockdown groups via flow cytometry in BXPC3 and SW1990 cell lines. **(E, F)** Percentage of CD24^+^ cells between the control group and the PCDH1 knockdown group **(G, H)** Percentage of CD133^+^ cells between the control group and the PCDH1 knockdown group. **(I, J)** Sphere formation assay was performed to detect sphere formation ability of the control group and the PCDH1 knockdown group in BXPC3 and SW1990. **(K, L)** Diameter of tumor spheres at 24 h, 48 h and 5 days in the control group and the PCDH1 knockdown group.(Scale bar = 500μm) (ns: *P*>0.05, **P < 0.01, ***P < 0.001, ****P < 0.0001).

To determine whether PCDH1 contributes to cancer stemness in PDAC, we examined its effect on canonical stemness markers and sphere-forming capacity. qRT-PCR analysis revealed that PCDH1 silencing markedly reduced the transcriptional levels of CD24 and CD133 in both BxPC3 and SW1990 cells (**Figures 10A, B**). Flow cytometric analysis confirmed a significant decline in CD24^+^ and CD133^+^ cell populations following PCDH1 knockdown (**Figures 10C–H**), indicating loss of stem-like subpopulations. Functionally, sphere formation assays further demonstrated that PCDH1-deficient cells exhibited impaired self-renewal ability. Both BxPC3 and SW1990 cells with PCDH1 silencing generated significantly smaller and fewer tumor spheres compared with controls, with differences becoming pronounced by day 5 of culture (**Figures 10I–L**). Taken together, these results provide compelling evidence that PCDH1 sustains stemness-associated phenotypes in PDAC, promoting the maintenance of CD24^+^/CD133^+^ subpopulations and enhancing self-renewal capacity.

### 3.10 Transcriptomic profiling reveals PI3K-Akt pathway involvement downstream of PCDH1

**Figure 11:**
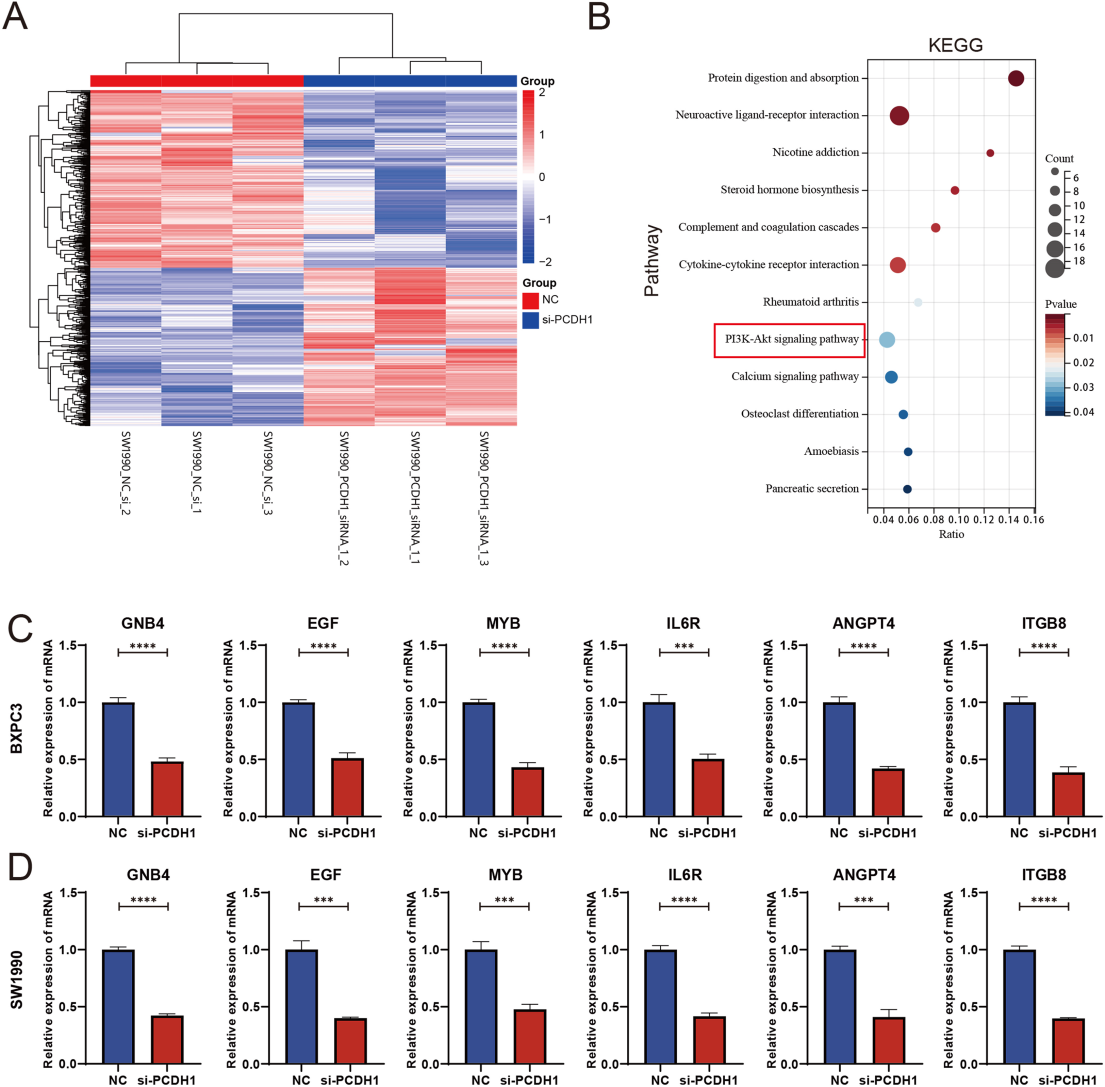
PCDH1 promotes proliferation and migration of PDAC through activation of PI3K/Akt pathway. **(A)** Heatmap of differential expression genes between the NC and si-PCDH1 groups. **(B)** KEGG enrichment analysis demonstrates pathways associated with differential expression genes. **(C, D)** Expression levels of mRNA for six molecules in the PI3K/Akt pathway were detected by qRT-PCR. (***P < 0.001, ****P < 0.0001).

To elucidate the molecular mechanisms underlying PCDH1 function in PDAC, transcriptomic profiling was performed following siRNA-mediated knockdown. Hierarchical clustering of differentially expressed genes revealed a distinct separation between control and PCDH1-silenced groups, with widespread transcriptional downregulation observed upon PCDH1 inhibition (**Figure 11A**). Subsequent KEGG pathway enrichment analysis highlighted significant involvement of cancer-related signaling networks, most prominently the PI3K-Akt pathway, alongside cytokine–cytokine receptor interactions and extracellular matrix–related processes (**Figure 11B**). To validate these findings, we examined the expression of representative downstream targets by qRT-PCR. Notably, genes implicated in oncogenic signaling and tumor progression, including GNB4, EGF, MYB, IL6R, ANGPT4, and ITGB8, were consistently downregulated in both BxPC3 and SW1990 cells following PCDH1 silencing (**Figures 11C, D**). Collectively, these data demonstrate that PCDH1 exerts its oncogenic role in PDAC at least partly by modulating PI3K-Akt signaling and associated tumor-promoting effectors.

### 3.11 IGF-1 rescues the proliferative impairment induced by PCDH1 knockdown

**Figure 12:**
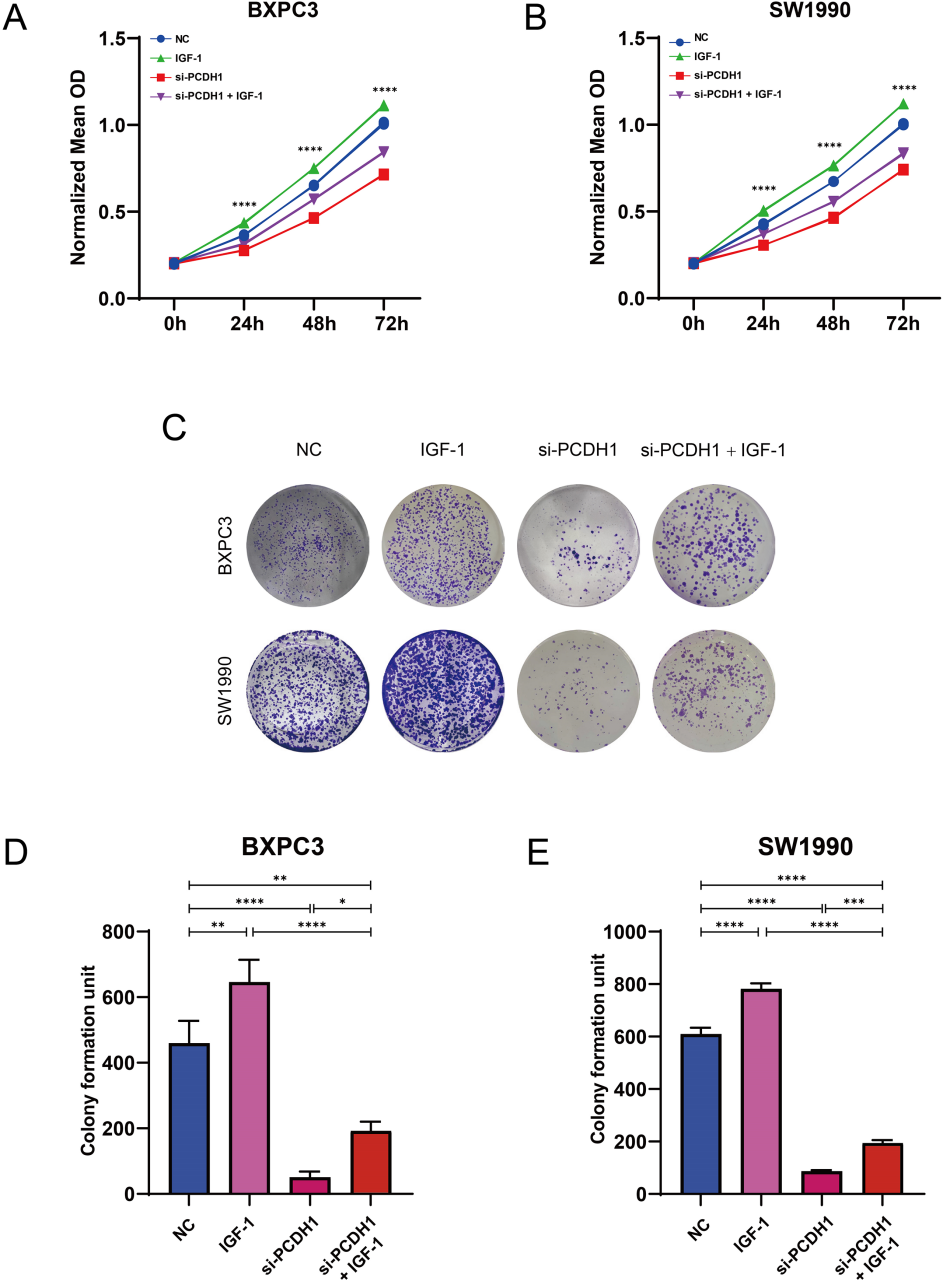
PCDH1 promotes proliferation and migration of PDAC through activation of PI3K/Akt pathway. **(A, B)** MTT assay was used to detect the proliferation ability of the control, IGF-1, si-PCDH1, and si-PCDH1 + IGF-1 groups. **(C–E)** A colony formation assay was performed to detect the proliferation ability of the four groups. (*P < 0.05, **P < 0.01, ***P < 0.001, ****P < 0.0001).

To explore whether PCDH1 exerts its oncogenic function via the PI3K/AKT axis, we conducted rescue assays using exogenous IGF-1 stimulation. MTT assays demonstrated that silencing PCDH1 markedly suppressed proliferation in both BxPC3 and SW1990 cells, whereas IGF-1 treatment significantly restored proliferative capacity. Notably, combined treatment with si-PCDH1 and IGF-1 partially rescued the growth defect, confirming the functional involvement of PI3K/AKT signaling (**Figures 12A, B**). Colony formation assays further corroborated these findings. PCDH1 knockdown drastically reduced clonogenic survival, while IGF-1 stimulation promoted robust colony formation. Importantly, IGF-1 partially reversed the inhibitory effect of si-PCDH1, though not to the level of IGF-1 stimulation alone, suggesting that PCDH1 may act upstream to potentiate PI3K/AKT-driven tumorigenicity (**Figures 12C–E**). Collectively, these results establish that PCDH1 promotes PDAC cell growth and clonogenicity, at least in part, through activation of the PI3K/AKT pathway, thereby functionally linking PCDH1 expression to oncogenic signaling cascades.

### 3.12 PCDH1 regulates pancreatic cancer cell migration

**Figure 13:**
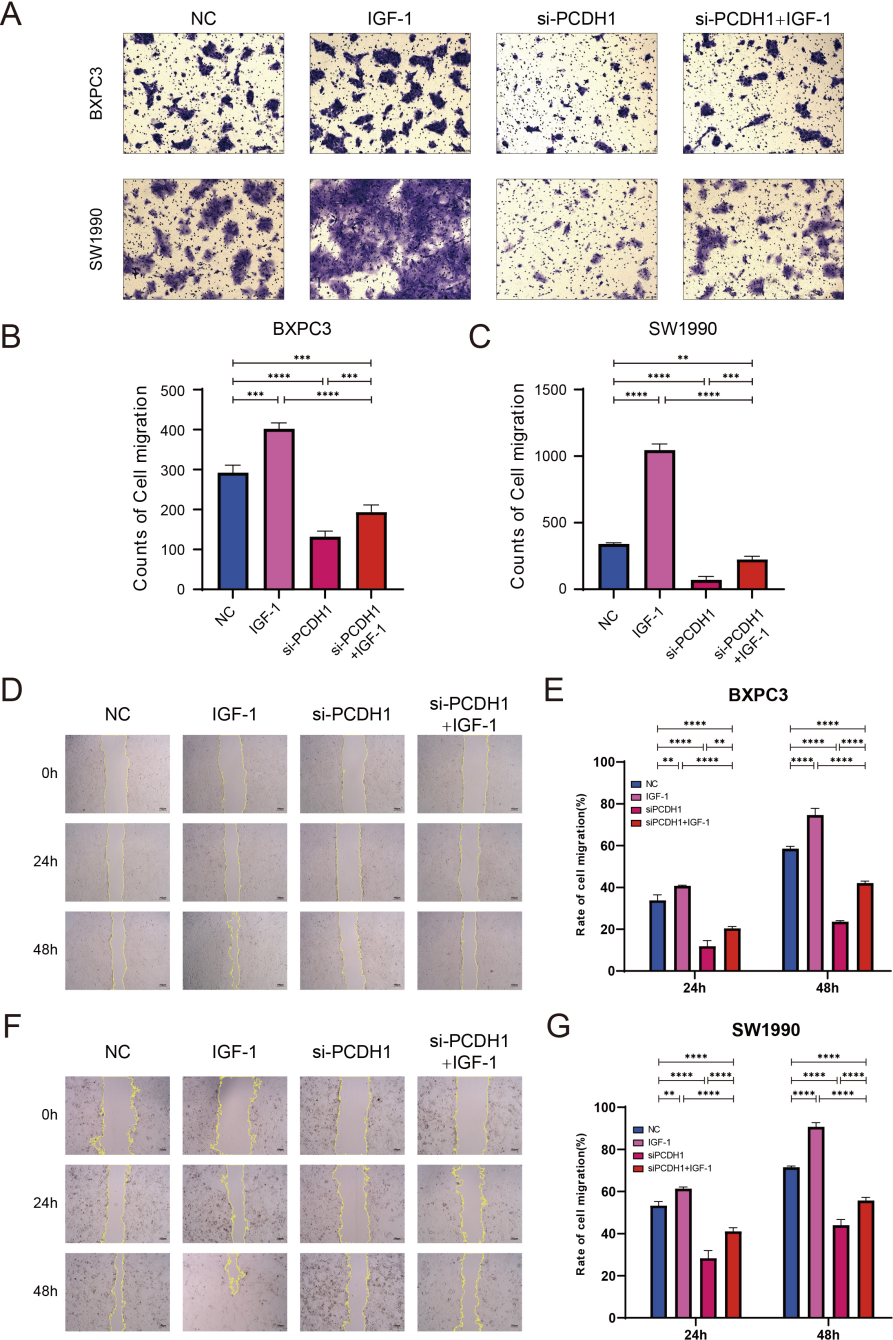
PCDH1 promotes proliferation and migration of PDAC through activation of PI3K/Akt pathway. **(A)** Transwell assays present the migration capacity of the control, IGF-1, si-PCDH1, and si-PCDH1 + IGF-1 groups. (Scale bar = 100μm) **(B, C)** Counts of cell migration in four groups. **(D, F)** Wound healing assays exhibit the migration ability of the four groups. (Scale bar = 100μm) **(E, G)** Rate of cell migration in four groups. (**P < 0.01, ***P < 0.001, ****P < 0.00011).

To investigate the effect of PCDH1 on cell motility, we first performed Transwell migration assays in BxPC3 and SW1990 cells. Knockdown of PCDH1 markedly reduced the number of migrated cells compared to the control group (P < 0.01). Conversely, stimulation with IGF-1 significantly enhanced migration. Notably, co-treatment with si-PCDH1 and IGF-1 partially restored migratory ability, although it remained lower than that of the IGF-1 group alone (**Figures 13A–C**). Consistent with the Transwell results, wound healing assays showed that PCDH1 silencing significantly delayed wound closure in both BxPC3 and SW1990 cells (P < 0.01). Cells treated with IGF-1 demonstrated accelerated wound closure, while the combination of si-PCDH1 and IGF-1 exhibited intermediate effects, indicating only partial rescue of the impaired migration (**Figures 13D–G**). Together, these findings demonstrate that PCDH1 knockdown suppresses pancreatic cancer cell migration, whereas IGF-1 stimulation counteracts this effect, suggesting that PCDH1 may regulate cell motility at least in part through the IGF-1–associated signaling pathway.

## 4 Discussion

Pancreatic ductal adenocarcinoma (PDAC) continues to rank among the most aggressive malignancies, characterized by rapid progression, early dissemination, and poor therapeutic response (23, 24). Despite advances in multimodal therapy, outcomes remain dismal, underscoring the urgent need to identify molecular determinants that sustain malignant behavior and therapeutic resistance. Increasing attention has been directed toward cancer stem cells (CSCs), a rare but functionally critical subset of tumor cells with self-renewal and multilineage potential (25, 26). CSCs not only initiate tumorigenesis but also fuel recurrence and metastasis by resisting cytotoxic and immune-mediated elimination. Our findings implicate protocadherin-1 (PCDH1) as a previously underappreciated regulator of stemness in PDAC (27).

Through integrative bioinformatics, we identified PCDH1 as a hub gene closely associated with stemness indices, and subsequent validation confirmed its overexpression in PDAC tissues compared with adjacent normal samples (28). Importantly, high PCDH1 expression correlated with reduced overall survival, highlighting its prognostic value (29–31). Functional assays demonstrated that PCDH1 promotes proliferative capacity, colony formation, and migratory potential, while knockdown substantially impaired these malignant traits (32). Moreover, stemness-related features, including spheroid formation and expression of canonical CSC markers CD24 and CD133, were markedly diminished upon PCDH1 silencing. These data collectively position PCDH1 as a driver of CSC maintenance in PDAC (33).

Mechanistically, transcriptomic profiling of PCDH1-deficient cells revealed downregulation of PI3K/AKT signaling, a pathway extensively linked to oncogenesis, metabolic adaptation, and therapeutic resistance in PDAC (34). Rescue experiments with IGF-1 further substantiated that PI3K/AKT activation is a critical mediator of PCDH1 function (35, 36). Thus, our study identifies PCDH1 not merely as a marker of stemness but as an upstream regulator of a canonical oncogenic cascade. Targeting this axis may therefore represent a rational strategy for CSC-directed therapy (37). Beyond tumor-intrinsic functions, PCDH1 expression exhibited strong correlations with an immunosuppressive tumor microenvironment (38). Elevated PCDH1 was associated with reduced infiltration of cytotoxic T cells, dendritic cells, and NK subsets, suggesting that PCDH1 may facilitate immune exclusion and tolerance (37). This dual role—sustaining CSC traits while dampening anti-tumor immunity—renders PCDH1 an especially compelling therapeutic candidate (39, 40). Furthermore, pharmacogenomic analyses indicated that PCDH1-high tumors display selective vulnerability to inhibitors of receptor tyrosine kinases and MAPK signaling, pointing toward opportunities for patient stratification in precision oncology (41, 42).

It should be acknowledged that this study also has certain limitations. This study was conducted primarily in 2D culture and awaits *in vivo* confirmation using organoids, patient-derived xenografts, or genetically engineered mouse models to validate PCDH1’s control of stemness and immune evasion. Detailed PI3K-AKT interactome mapping, immune-suppressive mechanisms, and drug-sensitivity predictions will require Co-IP/MS, phospho-proteomics, T-cell co-culture systems, and PDX efficacy assays. Future work will integrate multicenter clinical cohorts, single-cell multi-omics, and *in vivo* limiting-dilution transplantation to dissect the spatiotemporal PCDH1 signaling network and evaluate its translational potential as a companion diagnostic and a therapeutic target combinable with PI3K/mTOR inhibitors. In summary, our work establishes PCDH1 as a stemness-linked oncogene that orchestrates PDAC aggressiveness through PI3K/AKT activation while shaping immune evasion and drug response. These findings extend the biological understanding of PDAC stemness and highlight PCDH1 as both a prognostic biomarker and a potential therapeutic target. Future investigations should focus on validating these observations in preclinical models, exploring the structural basis of PCDH1–PI3K/AKT crosstalk, and assessing its utility as a companion biomarker in clinical trials.

## 5 Conclusion

Our study identifies PCDH1 as a stemness-linked oncogene in pancreatic ductal adenocarcinoma (PDAC). Integrative analyses revealed that PCDH1 is markedly upregulated in tumors, correlates with poor patient survival, and drives malignant phenotypes including proliferation, migration, and cancer stem cell maintenance. Mechanistically, PCDH1 promotes PDAC progression through activation of the PI3K/AKT signaling cascade, thereby coupling stemness traits with oncogenic signaling. In addition, elevated PCDH1 expression was associated with immune exclusion and selective drug sensitivities, highlighting its relevance not only as a prognostic biomarker but also as a potential determinant of therapeutic response. Taken together, these findings position PCDH1 at the intersection of stemness regulation, tumor aggressiveness, and treatment resistance in PDAC. By linking CSC biology to immune modulation and actionable signaling pathways, PCDH1 offers a promising target for precision oncology approaches. Future work should validate these observations in *in vivo* models and assess the translational potential of PCDH1-directed interventions, both as a therapeutic target and as a stratification marker in clinical management of PDAC.

## Data Availability

The original contributions presented in the study are included in the article/supplementary material. Further inquiries can be directed to the corresponding authors.

## Glossary

AKT: Protein kinase B
ANGPT4: Angiopoietin-4
APC: Antigen-presenting cell
AUC: Area under the curve
bFGF: Basic fibroblast growth factor
B3GNT3: β-1,3-N-acetylglucosaminyltransferase 3
BSA: Bovine serum albumin
CI: Confidence interval
CSC: Cancer stem cell
CTRP: Cancer Therapeutics Response Portal
DAB: 3,3′-Diaminobenzidine
DEG: Differentially expressed gene
DMSO: Dimethyl sulfoxide
DNAss: DNA-methylation-based stemness score
EGF: Epidermal growth factor
ESRP1: Epithelial splicing regulatory protein 1
ESTIMATE: Estimation of STromal and Immune cells in MAlignant Tumor tissues using Expression data
FBS: Fetal bovine serum
FFPE: Formalin-fixed paraffin-embedded
FDR: False discovery rate
FITC: Fluorescein isothiocyanate
FPKM: Fragments per kilobase of transcript per million mapped reads
GDSC: Genomics of Drug Sensitivity in Cancer
GNB4: Guanine nucleotide-binding protein subunit β-4
GSDC: Gene Set Differential Correlation
HR: Hazard ratio
HRP: Horseradish peroxidase
IC50: Half-maximal inhibitory concentration
IGF-1: Insulin-like growth factor-1
IL6R: Interleukin-6 receptor
IOD: Integrated optical density
IHC: Immunohistochemistry
ITGB8: Integrin β-8
KCNK1: Potassium two-pore domain channel subfamily K member 1
KEGG: Kyoto Encyclopedia of Genes and Genomes
Limma: Linear models for microarray data
MAL2: MAL proto-oncogene, T-cell differentiation protein 2
MOD: Mean optical density
mRNAsi: mRNA-based stemness index
MTT: 3-(4,5-Dimethylthiazol-2-yl)-2,5-diphenyltetrazolium bromide
MYB: MYB proto-oncogene, transcription factor
NK: Natural killer
ns: Not significant
OS: Overall survival
PAAD: Pancreatic adenocarcinoma
PBS: Phosphate-buffered saline
PCR: Polymerase chain reaction
PDAC: Pancreatic ductal adenocarcinoma
PCDH1: Protocadherin-1,PE:Phycoerythrin
PERP: p53 apoptosis effector related to PMP-22
PI: Propidium iodide
PI3K: Phosphatidylinositol 3-kinase
PPI: Protein–protein interaction
pDC: Plasmacytoid dendritic cell
qRT-PCR: Quantitative real-time PCR
RNAss: RNA-based stemness score
ROC: Receiver operating characteristic
RPMI: Roswell Park Memorial Institute medium
RT: Room temperature
SD: Standard deviation
SDRC5: Short-chain dehydrogenase/reductase family 16C member 5
SEM: Standard error of the mean
si-NC: Small interfering negative control
si-PCDH1: Small interfering RNA targeting PCDH1
SMAD3: SMAD family member 3
ssGSEA: Single-sample gene set enrichment analysis
TCGA: The Cancer Genome Atlas
TFH: T follicular helper
TISCH: Tumor Immune Single-cell Hub
TMPRSS4: Transmembrane protease serine 4
TOB1: Transducer of ERBB2-1
Treg: Regulatory T cell
UCSC: University of California, Santa Cruz
WGCNA: Weighted gene co-expression network analysis.
